# From Phytochemistry to Oncology: The Role of Bakuchiol in the Treatment of Breast Cancer

**DOI:** 10.3390/biom16010094

**Published:** 2026-01-06

**Authors:** Magdalena Czarnecka-Czapczyńska, David Aebisher, Alina Pietryszyn-Bilińska, Magdalena Moś, Sara Czech, Jakub Szpara, Dorota Bartusik-Aebisher, Aleksandra Kawczyk-Krupka

**Affiliations:** 1 Department of Internal Diseases, Angiology and Physical Medicine, Center for Laser Diagnostics and Therapy, Medical University of Silesia, Batorego 15, 41-902 Bytom, Poland; magdalena.czarnecka921114@gmail.com (M.C.-C.); a.pietryszynbilinska@gmail.com (A.P.-B.); 2 Departement of Photomedicine and Physical Chemistry, Collegium Medicum, Faculty of Medicine, Rzeszów University, 35-310 Rzeszów, Poland; 3 Specialist Hospital No. 2, Department of Internal Diseases, Angiology and Physical Medicine, Center for Laser Diagnosis and Therapy, Batorego Street 15, 41-902 Bytom, Poland; magdamos01@interia.pl; 4 English Division Science Club, Collegium Medicum, Faculty of Medicine, 35-310 Rzeszów, Poland; sc126240@stud.ur.edu.pl (S.C.); js126214@stud.ur.edu.pl (J.S.); 5 Department of Biochemistry and General Chemistry, Collegium Medicum, Faculty of Medicine, Rzeszów University, 35-310 Rzeszów, Poland; dbartusikaebisher@ur.edu.pl

**Keywords:** bakuchiol, breast cancer, molecular mechanism, molecular pathways, bakuchiol extraction, bakuchiol in treatment

## Abstract

Bakuchiol (BAK), a natural meroterpenoid with antioxidant, anti-inflammatory and anticancer properties, has recently gained attention as a potential adjunct in breast cancer therapy. This review contextualizes breast cancer as a major global health challenge and highlights BAK as a bioactive compound capable of modulating pathways relevant to tumor development and progression. A structured literature search identified studies examining its molecular activity, pharmacological profile, and effects on breast cancer cells and stem cells. Results show that BAK influences oxidative stress regulation, mitochondrial function, apoptosis and estrogen receptor signaling while also affecting PI3K/AKT, MAPK, NF-κB, and EMT-related pathways. In breast cancer models, BAK acts as a selective phytoestrogen, induces S-phase arrest, activates the ATM/ATR–Chk1/Chk2 axis, and triggers mitochondrial apoptosis, particularly in ERα-positive cells. It also suppresses breast cancer stem-cell renewal, promotes BNIP3- and DAPK2-mediated apoptosis, reduces metabolic and transcriptional drivers of metastasis, and shows enhanced anticancer activity in derivative forms. These findings suggest that BAK may provide therapeutic benefit across several mechanisms central to breast cancer biology. In this review, the inclusion criteria encompassed publications describing the action of bakuchiol, its chemical and pharmacological properties, as well as its role in the treatment of various conditions, including cancers. Exclusion criteria included works not related to BAK or its therapeutic use in breast cancer, as well as publications that did not meet basic scientific standards, such as lacking methodological rigor or presenting a low level of scientific evidence. However, current evidence is predominantly in vitro, and limitations such as poor bioavailability and lack of clinical validation underscore the need for further in vivo and translational studies before therapeutic application can be established.

## 1. Introduction

### 1.1. Epidemiology

**Figure 1:**
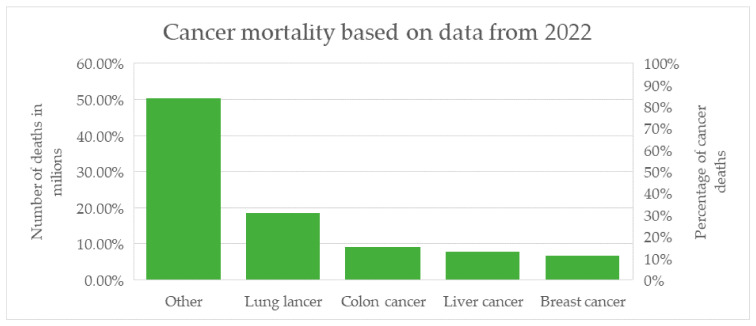
Summary of the most common causes of cancer-related deaths worldwide based on 2022 data, including the percentage share and the number of deaths for selected cancer types [1,2,3,4].

The vast majority of breast cancer cases—approximately 99%—occur in women [1]. Globally, this cancer is among the most frequently diagnosed in women and represents one of the leading causes of cancer-related deaths. In recent decades, higher incidence rates have been observed in highly developed countries; however, these regions also report better prognoses, which result from easier access to specialized medical care, screening programs, and modern treatment methods. This is confirmed by mortality data, amounting to 11.3 vs. 15.3 per 100,000 women in developed and developing countries, respectively [2]. The most recent global mortality statistics come from reports based on 2022 data and published in early 2024 by the American Cancer Society (ACS) and the International Agency for Research on Cancer (IARC), as presented in Figure 1.

The introductory section presents the epidemiology of breast cancer and a general characterization of bakuchiol (BAK), including its chemical structure, natural sources, and known biological properties. Subsequent sections focus on the current state of knowledge regarding the molecular basis of BAK activity and its potential therapeutic role in breast cancer treatment. Particular attention was devoted to the modulatory effects of bakuchiol on signaling pathways associated with breast carcinogenesis, indicating its considerable potential as a bioactive compound with anticancer properties.

Key risk factors for breast cancer include environmental and lifestyle-related elements such as hormone exposure, the use of psychoactive substances, diet, and physical activity levels. Factors independent of the patient also play an important role, including age, race, age at menarche, and genetic predisposition. Mutations in the BRCA1 and BRCA2 genes account for approximately 10% of all breast cancer cases [3].

Current therapeutic strategies include surgical methods such as lumpectomy, mastectomy, and axillary lymph node dissection, as well as systemic treatments: radiotherapy, chemotherapy, hormone therapy, targeted therapy, immunotherapy, and—in advanced stages—palliative care [4]. Ongoing research focuses on developing therapies that are more effective, safer, and based on natural compounds, which may be associated with fewer adverse effects compared to conventional methods.

### 1.2. Bakuchiol (BAK)

Bakuchiol (C_18_H_24_O) is a natural meroterpenoid phytocompound with a broad range of biological activities. Since its identification in 1966, this compound and its analogs have found applications in both medicine and cosmetology [5]. The primary source of BAK is the seeds and leaves of *Psoralea corylifolia*, a plant long used in dermatology [6]. However, the compound is also present in other plant species, which are listed in Table 1.

BAK exhibits remarkable diversity in both its structural and chemical nature. In plant organisms, its synthesis occurs through a mixed biosynthetic pathway that combines amino acids and isoprene. As a result, molecular chemical modifications take place, involving stereogenic centers and various functional groups, which confer the compound’s unique properties [5].

BAK demonstrates numerous pharmacological properties, including antioxidant and anti-inflammatory activities [19]. Its antioxidant activity results from the inhibition of lipid peroxidation induced by tBH, CCl_4_, D-GalN, and NADH, as well as from the neutralization of reactive oxygen species and free radicals such as Cl_3_CO_2_•, LOO•, DPPH•, and the hydroxyl radical [20]. The anti-inflammatory properties of bakuchiol are associated with the reduction of inflammatory mediators, including NO, PGE_2_, LTB_4_, and TxB_2_ [19,20]. The compound also exhibits antiallergic [21] and hepatoprotective effects [22], manifested, among other mechanisms, by the induction of apoptosis in hepatic stellate cells (HSCs) through a caspase-3–dependent pathway. Its cardioprotective effects have also been described, involving increased SIRT1 expression, elevated Bcl-2 levels, and decreased caspase-3 and Bax expression, which protects cardiomyocytes from ischemia–reperfusion injury [23]. Moreover, BAK positively influences extracellular matrix metabolism—it inhibits MMP-1 expression and increases TIMP-2 levels, supporting collagen synthesis and limiting its degradation, which imparts anti-aging properties [24]. Additional reported activities include antidepressant and antistress effects [25,26].

BAK also acts as a phytoestrogen capable of modulating estrogen receptors. Depending on its concentration, it may function as an agonist or antagonist of ERs, including ERα [20,27]. In contrast to conventional hormone replacement therapy, it has been suggested to have a different safety profile, which may be of relevance in the context of hormone-dependent cancers.

Another important characteristic of bakuchiol is its antibacterial activity, especially against microorganisms of the oral cavity, such as *Streptococcus mutans, S. sanguis, S. sobrinus, Enterococcus faecalis, Lactobacillus plantarum, Actinomyces viscosus*, and *Porphyromonas gingivalis* [20].

**Figure 2:**
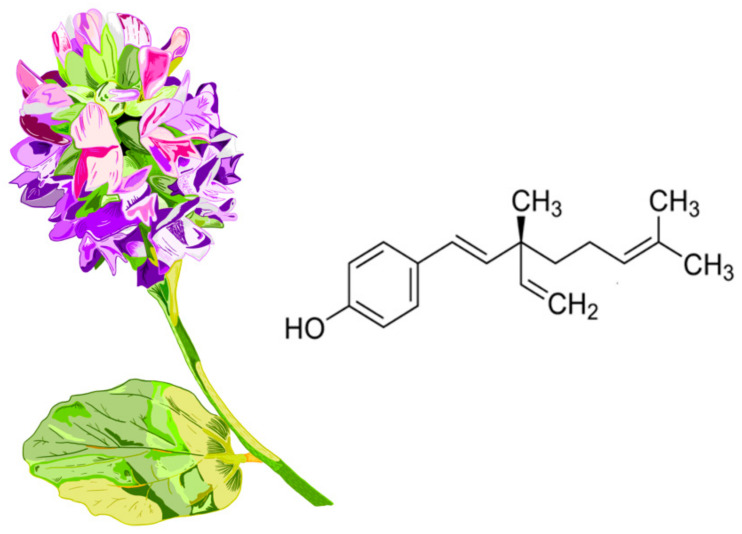
Chemical structure of bakuchiol (formally named 4-[(1E,3S)-3-ethenyl-3,7-dimethylocta-1,6-dienyl]phenol). The appearance of the plant *Psoralea corylifolia*. The plant produces small bluish-purple or yellow flowers arranged in dense clusters (racemes). Bakuchiol is a natural derivative primarily from the seeds of the *Psoralea corylifolia* plant. The seeds are brownish-black, kidney-shaped (reniform), and have a bitter, aromatic odor.

Chemically, BAK represents a structural fusion of phenolic and terpenoid elements. The molecule contains an aromatic phenolic ring with a hydroxyl group and an unsaturated hydrocarbon chain with three double bonds and a fully substituted quaternary center, classifying it as a meroterpenoid [28]. Considering its long hydrophobic chain, BAK exhibits low solubility in H_2_O and poor bioavailability. Even more interestingly, it undergoes extensive first-pass metabolism, undoubtedly influenced by its propensity to form covalent bonds with endogenous molecules via the hydroxyl group of the phenolic acid present in its structure [5,29]. The chemical structure of the compound and the appearance of the plant *Psoralea corylifolia*, from which this compound is naturally derived, are shown in Figure 2.

Initially, the natural synthesis of bakuchiol posed a considerable challenge; however, researchers have gradually improved the methods, and a compilation of their most significant achievements and the available natural extraction techniques is presented chronologically in Table 2 below.

There is also the possibility of isolating BAK through chemical synthesis, with the earliest attempts dating back to 1967, when Damodaran and Dev described the methyl ether of racemic BAK. This was the first chemical synthesis of the compound, and it is worth noting that it proceeded in three steps [28]. The first enantioselective synthesis of (+)-BAK was achieved in 1990 via a two-step pathway [37]. Regarding the method for obtaining (S)-BAK and (R)-BAK, a breakthrough occurred in 2008, when the former was synthesized through a ten-step route and the latter through a nine-step route [38]. In terms of the most recent advances, special attention should be given to the 2020 report in which racemic BAK was synthesized using a method based on regioselective molybdenum-catalyzed reactions combined with allylic substitution [39].

Despite BAK’s low toxicity and satisfactory in vitro effects, its effectiveness in clinical trials with patients may be significantly limited. This may be due to BAK’s poor water solubility, which limits its absorption in the gastrointestinal tract and, consequently, its concentration in the body [40]. It also undergoes first-pass metabolism in the liver via cytochrome P450 [41]. Rapid metabolism and excretion shorten its time in the body, which reduces its concentration in healthy tissues and tumors. Bakuchiol is unstable in an acidic environment, which limits its concentration in gastric juice (pH 1.5–3.5), cervical mucus (pH 4.0–5.0), sweat (pH 4.5–6.0), breast milk (pH 6.5–7.0) and intercellular fluid in skin (pH 4.5–5.5) [42]. Therefore, a concentration of ≥4–7 μg/mL that is successfully used in vitro may not be achievable in the human body [43].

Given the growing interest in natural compounds as complementary elements of modern oncological therapy, BAK stands out as a molecule with multidirectional biological potential. Current findings suggest that it may influence processes essential for tumor development and progression, making it an intriguing candidate for further evaluation in the context of supportive cancer therapies. Therefore, the aim of this review is to provide a concise overview of the current knowledge on BAK, with particular emphasis on its potential role in the treatment of breast cancer and other malignancies, as well as to identify areas requiring further research. Such an approach makes it possible to place BAK within the broader context of contemporary oncology and to highlight its potential value as a natural compound with therapeutic relevance.

Our review presents a new perspective on bakuchiol and phytochemicals in breast cancer treatment by shifting focus from general anticancer properties to targeted molecular mechanisms and advanced delivery systems. While older reviews focused on broad cytotoxicity, 2025 perspectives highlight bakuchiol’s specific ability to target Breast Cancer Stem Cells (BCSCs) [44]. Bakuchiol has been shown to repress Notch3 expression, which is critical for the self-renewal capacity of BCSCs. New research utilizes zebrafish xenografts to demonstrate bakuchiol’s ability to inhibit in vivo metastasis by upregulating CK18 and downregulating factors like FASN and TGFBR1 [45]. Recent reviews emphasize the synergistic potential of combining bakuchiol with other phytochemicals or standard treatments to overcome drug resistance. A 2025 review identifies a specific synergy between bakuchiol and isobavachalcone, which together inhibit DNA replication and the CHK2 signaling pathway to prevent tumor development. There is a new emphasis on integrating these compounds as fundamental parts of all-encompassing cancer treatment rather than just dietary supplements.

## 2. Materials and Methods

Work on this literature review began with a systematic analysis of scientific publications retrieved from the PubMed and Google Scholar databases. The literature search covered publications published from 1965 onward. To ensure a high level of reliability in the review process, review articles, in vitro and in vivo studies, as well as clinical research related to bakuchiol (BAK), were included, both in the context of its general biological properties and its potential application in breast cancer therapy. The literature search was conducted using the following keywords: bakuchiol, breast cancer, molecular mechanism, molecular pathways, bakuchiol extraction, and bakuchiol in treatment. The inclusion criteria encompassed publications describing the action of bakuchiol, its chemical and pharmacological properties, as well as its role in the treatment of various conditions, including cancers. Exclusion criteria included works not related to BAK or its therapeutic use in breast cancer, as well as publications that did not meet basic scientific standards, such as lacking methodological rigor or presenting a low level of scientific evidence. The first stage of the analysis involved selecting specialist articles that met the predefined criteria. In total, 267 studies were initially identified and screened. After applying the inclusion and exclusion criteria and conducting a detailed qualitative assessment, 73 publications were ultimately included in the final analysis. Publications that passed the initial screening were subjected to detailed scientific evaluation, with particular attention given to the molecular mechanisms of bakuchiol activity in various disease models. Special emphasis was placed on the potential influence of BAK on key signaling pathways implicated in the pathogenesis of breast cancer, including mechanisms regulating proliferation, apoptosis, oxidative stress, and hormonal receptor modulation. Several essential thematic areas were identified to structure the review. The introductory section presents the epidemiology of breast cancer and a general characterization of bakuchiol, including its chemical structure, natural sources, and known biological properties. Subsequent sections focus on the current state of knowledge regarding the molecular basis of BAK activity and its potential therapeutic role in breast cancer treatment. Particular attention was devoted to the modulatory effects of bakuchiol on signaling pathways associated with breast carcinogenesis, indicating its considerable potential as a bioactive compound with anticancer properties.

## 3. Results

### 3.1. Molecular Mechanisms of BAK

A breast cancer cell forms when a healthy cell accumulates mutations in genes that control cell growth and death. Mutagens can be factors that damage the cell or its individual structures. These include chemicals, viruses and chronic inflammation. These mutations activate oncogenes and disable tumor suppressor genes, leading to uncontrolled division, evasion of apoptosis, and genetic instability, ultimately leading to cancer development.

Few viruses and bacteria are oncogenic, but most untreated infections can result in chronic inflammation. In in vitro studies of Madin-Darby canine kidney (MDCK) cells infected with the H1N1 virus, BAK reduced viral RNA and protein levels, inhibiting viral gene expression while simultaneously enhancing the antiviral response [46]. Furthermore, BAK inhibited SARS-CoV-2 entry and blocked the binding of the RBD-ACE2 complex, without demonstrating toxicity [46,47]. BAK and its derivatives act mainly by permeabilizing bacterial membranes, which leads to a rapid loss of bacterial cell viability and disruption of the biofilm [48]. Furthermore, BAK and its derivatives can act as antibiotic adjuvants by inhibiting efflux pumps, destabilizing membranes, and also disrupting biofilms [49,50]. Studies have shown that BAK exerts anti-inflammatory effects by inhibiting PGE_2_ and IL-6 in cells, as well as iNOS and COX-2 expression without cytotoxicity. The mechanism involves inhibition of the p38, MAPK, and ERK pathways and suppression of NF-κB and STAT3, leading to a decrease in the production of inflammatory mediators [51,52]. BAK achieves its anti-inflammatory effect by increasing NO inhibition through increased iNOS expression while reducing cytotoxicity [53]. Collectively, the results confirm that BAK suppresses inflammation by inhibiting NF-κB and MAPK pathways and limiting cytokine and ROS production [54].

Research indicates that the terpenoid chains present in BAK provide protection against lipid peroxidation. BAK protects the activity of mitochondrial respiratory enzymes against NADPH-dependent and dihydroxyfumarate-induced peroxidative damage and additionally exhibits a protective effect on mitochondria exposed to oxidative stress, thus increasing cell viability [55]. Studies in rats have shown that BAK induces caspase-dependent apoptosis of activated hepatic stellate cells by increasing oxidative stress and activating the JNK pathway. This leads to a mitochondrial apoptotic cascade (Bax, cytochrome c, caspase-3, PARP), which promotes the selective elimination of these cells and may contribute to the reversal of liver fibrosis [56]. Additionally, BAK displayed protective effects on retinal cells exposed to oxidative injury [57]. In vivo and in vitro in cardiomyocytes, BAK reduces inflammation by limiting oxidative stress and inhibiting NF-κB activation [58]. BAK protects cardiomyocytes against the effects of ischemia by improving mitochondrial function and regulating oxidative stress dependent on the SIRT1/PGC-1α pathway, which limits apoptosis and cell damage [23]. BAK has anti-aging effects by increasing the expression of collagen I and III and TIMP-1/2, while reducing MMP-1, which is associated with an increase in the viability of ESF-1 cells [59].

#### 3.1.1. General Anticancer Mechanism

Recent studies have identified several ways in which BAK might combat breast cancer cells. However, based on the literature, BAK combats breast cancer through three primary mechanisms [6,60]:

Firstly, BAK targets Breast Cancer Stem Cells (BCSCs). It achieves this by inhibiting mammosphere formation, preventing the clustering of stem-like cells, and/or reducing ALDH (aldehyde dehydrogenase) activity, a key functional marker for cancer stemness.

Secondly, BAK induces cell cycle arrest and apoptosis. It achieves this by halting the proliferation of cancer cells by disrupting their division cycle and triggering programmed cell death. Specifically, it triggers S-phase arrest, thereby preventing DNA replication and effectively stopping cell division. BAK increases the phosphorylation of Cdc2 (Tyr15) by activating kinases Myt1 and Wee1, which serve as a molecular “brake” on the cell cycle.

Thirdly, BAK inhibits metastasis and Epithelial–Mesenchymal Transition (EMT). It achieves this by suppressing the ability of breast cancer cells to spread (metastasize) and undergo the EMT process, where cells become mobile and invasive. BAK downregulates pro-invasive genes by reducing the expression of Notch3, FASN, TGFBR1, and ACVR1B. BAK promotes a less aggressive cell state by increasing markers like CK18 and E-cadherin, which help cells stay adhered to one another rather than migrating.

#### 3.1.2. General Estrogenic Effect

BAK is a phytoestrogen, meaning it can mimic or modulate estrogen in the body [61,62,63]. This creates a “biphasic effect” that requires caution:

1. Low-Dose Stimulation: Some research indicates that at very low concentrations, bakuchiol may actually stimulate the growth of ERα-positive breast cancer cells.
2. High-Dose Inhibition: Conversely, at higher concentrations, it appears to inhibit growth and suppress estrogen receptor alpha (ERα) while inducing estrogen receptor beta (ERβ), which is often associated with tumor suppression.

BAK shows complex estrogenic effects: it can act like estrogen (phytoestrogen) at low doses, potentially stimulating some hormone-sensitive cells, but also exhibits significant anticancer, antiproliferative, and anti-inflammatory actions, especially at higher doses or in specific contexts like benign prostatic hyperplasia (BPH) or breast cancer models, by affecting estrogen receptor (ER) types (ERα/ERβ) and other pathways; however, because it does have estrogen-like activity, those with serious hormone-sensitive conditions (like hormone-receptor-positive cancers) should consult a doctor due to potential risks, though it is often seen as a gentler retinoid alternative [63].

### 3.2. BAK in the Treatment of Breast Cancer

There are multiple mechanisms by which bakuchiol modulates cellular signaling pathways; these have been collected and systematically organized in Table 3, with classification into inflammation, angiogenesis, cell cycle, apoptosis and autophagy [64].

Evidence of the potential of BAK in breast cancer treatment appeared as early as 2010. Most importantly, different fractions of the PCL extract selectively activated estrogen receptor subtypes ERα and ERβ. It was shown that BAK acted as an agonist of both ERα and ERβ, and all of its effects were blocked by the estrogen receptor antagonist ICI 182,780, confirming that it acts directly through estrogen receptors [68]. In the context of breast cancer, it is particularly relevant that, unlike selective ERα agonists such as psoralen and isopsoralen, BAK did not increase the proliferation of MCF-7 cells despite its ER activity. This may be due to its simultaneous activation of ERβ, which is known to exert antiproliferative effects by suppressing the transcription of growth-promoting genes induced by ERα [69]. In the same year, the in vitro anticancer activity of BAK, previously demonstrated in other models, was evaluated in comparison with tamoxifen. The biological activity profile showed that BAK can inhibit the growth of human breast cancer, with IC_50_ values of 2.89 × 10^−5^ mol L^−1^ and 8.29 × 10^−3^ mol L^−1^ against the T-47D and MDA-MB-231 cell lines, respectively [67]. In subsequent years, researchers reported that at higher concentrations of BAK (≥4–7 μg/mL), its activity profile changes. Specifically, BAK decreases ERα expression and increases ERβ expression in MCF-7 cells. Functionally, this shifts its overall effect toward the antiproliferative role of ERβ and gives it the characteristics of a selective estrogen receptor modulator. At the cellular level, BAK induces S-phase arrest in both MCF-7 and ERα-negative MDA-MB-231 cells. This is accompanied by increased phosphorylation of Cdc2 (CDK1) at Tyr15, elevated expression of the kinases Wee1 and Myt1, and increased levels of cyclin B1, indicating that the Cdc2/cyclin B1 complex remains in an inactive form and cells are unable to proceed to mitosis. Furthermore, BAK induces p21^Waf1/Cip1 expression and, at the mRNA level, upregulates ATM kinase, with subsequent activation of the checkpoint kinases Chk1 and Chk2. Reversal of S-phase arrest and reduced Chk1/Chk2 activation following caffeine treatment (an ATM/ATR inhibitor) indicates that BAK triggers the ATM/ATR → Chk1/Chk2 → Cdc25/Cdc2 axis, typical of the response to DNA damage or replication block. A second anticancer mechanism described in the study was the induction of mitochondrial apoptosis, whose key event is the loss of mitochondrial membrane potential. This is accompanied by increased levels of proapoptotic Bcl-2 family proteins Bim, Bax, and Bak and activation of caspase-9 and caspase-7, leading to PARP cleavage. Notably, apoptosis occurs predominantly in ERα-positive MCF-7 cells, while in ERα-negative MDA-MB-231 cells, BAK mainly induces S-phase arrest, suggesting that the presence of ERα and modulation of the ERα/ERβ ratio enhances the apoptotic response [70]. In a study focused on breast cancer stem cells (BCSCs; CD44^+^/CD24^−^/low), the effect of BAK on this population was examined. Functionally, BAK inhibited their self-renewal capacity—it reduced both the number and size of mammospheres in unsorted MCF-7 cells as well as in the isolated CD44^+^/CD24^−^/low fraction and decreased ALDH activity, a classical stemness marker. BAK induced apoptosis in BCSCs via the mitochondrial pathway. Specifically, it increased the proportion of early apoptotic cells, reduced mitochondrial membrane potential, and significantly upregulated the proapoptotic genes BNIP3 and DAPK2. BNIP3, as a BH3-only protein, links mitochondrial stress, ROS generation, and Δψm loss with activation of the intrinsic apoptotic pathway—thus, the combined profile of elevated BNIP3/DAPK2 expression and decreased Δψm indicated mitochondrial initiation of the death program in cancer stem cells. Additionally, BAK increased ROS production in a dose-dependent manner, and at higher concentrations this exceeded the oxidative tolerance of BCSCs, thereby triggering apoptosis. At the same time, high concentrations of BAK inhibited FASN activity and reduced lipid synthesis, depriving cells of a key energy source and limiting processes that favor metastasis. Moreover, BAK reprogrammed the metastasis-associated transcriptional profile: it increased CK18 expression while decreasing Notch3, TGF-β receptors, and FASN levels in BCSCs. The TGF-β–Notch3–FASN axis is crucial for epithelial–mesenchymal transition (EMT), a process in which epithelial cells lose their stable, adherent phenotype and acquire migratory, invasive properties. Its suppression by BAK therefore simultaneously weakens stem-like features, migratory capacity, and metabolic adaptations that normally facilitate metastatic spread [70].

A 2024 study described a new BAK derivative, bakuchiol aminoguanidine, which induced cell death in triple-negative breast cancer MDA-MB-231 cells via a clearly mitochondrial apoptotic pathway. In this case, the effect was driven by increased expression of proapoptotic proteins such as cytochrome c, caspase-3, and Bax, together with reduced Bcl-2 levels, shifting the balance toward signaling that compromises mitochondrial integrity. This led to a decrease in Δψm, confirming mitochondrial damage, and to enhanced ROS production, which further amplified proapoptotic signals. Together, these changes indicate that this derivative induces apoptosis through mitochondrial destabilization and activation of the intrinsic apoptotic cascade [67].

**Figure 3:**
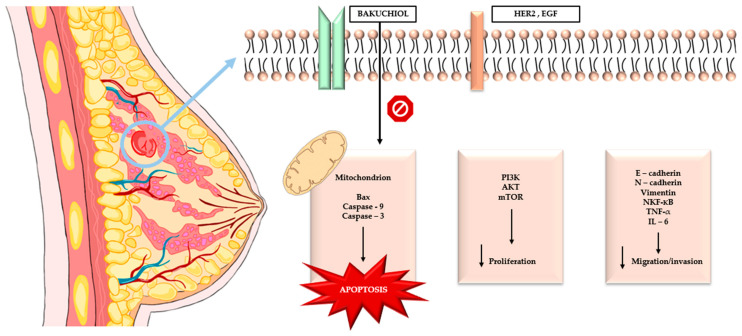
The graphical summary of the cellular pathways through which bakuchiol exerts its effects in breast cancer.

In 2025, nineteen new BAK derivatives were investigated; among them, compound 19 showed the strongest activity against MDA-MB-231 breast cancer cells. The primary mechanism of compound 19 is apoptosis induction via activation of the mitochondrial cell-death pathway. At the molecular level, this is based on increased expression of the proapoptotic protein Bax and decreased expression of the antiapoptotic protein Bcl-2, resulting in an elevated Bax/Bcl-2 ratio and mitochondrial membrane destabilization. Consequently, cytochrome c is released into the cytoplasm, and caspase-3—the key executioner enzyme of apoptosis—is activated. Inhibition of apoptosis by the pan-caspase inhibitor z-VAD-fmk confirms that the action of compound 19 is caspase-dependent. In addition to promoting cell death, compound 19 effectively suppresses the migratory and invasive capacity of MDA-MB-231 cells by modulating metastasis-related pathways. It reduces the expression of MMP-2 and MMP-9, which are responsible for extracellular matrix degradation, while increasing E-cadherin levels, a key protein maintaining cell–cell adhesion and inhibiting EMT. As a result, cancer cells lose the ability to actively migrate and invade surrounding tissues. Its efficacy was also confirmed in vivo: in an MDA-MB-231 xenograft model, compound 19 significantly inhibited tumor growth without causing notable toxic effects [71]. The graphical summary of the cellular pathways through which bakuchiol exerts its effects in breast cancer is presented in Figure 3. Table 4 presents comparative summarized information on breast cancer cell lines used, ER/HER2 status, bakuchiol concentrations and primary outcomes.

## 4. Discussion

The data gathered in this review indicate that BAK is a multidirectional bioactive compound whose numerous mechanisms of action overlap with key pathways involved in the development of breast cancer. Findings from studies describing its antiviral, antioxidant, anti-inflammatory, antibacterial and anticancer properties show a consistent pattern of modulation of oxidative stress, mitochondrial integrity, apoptotic pathways and transcriptional regulators. These play a crucial role in the proliferation, survival, metastasis and treatment resistance of breast cancer cells, providing a rationale for further investigation of BAK as a potential breast anticancer agent. BAK is a lipophilic meroterpene derived primarily from the seeds of *Psoralea corylifolia*. Its pharmacokinetic profile is characterized by low oral bioavailability and slow elimination. Recent 2025 research indicates that formulations with polyvinylpyrrolidone (PVP) or nanocarriers like micelles and nanoemulsions can significantly improve its solubility and bioavailability [72]. BAK is currently being investigated in preclinical studies as a potential anti-breast cancer agent, though it is not yet an approved treatment. Research primarily focuses on its ability to inhibit cancer cell growth, target cancer stem cells, and work synergistically with other therapies. Bakuchiol has been shown to sensitize certain cancer cells to TRAIL-induced apoptosis by upregulating death receptors (DR4 and DR5) through the ROS/JNK pathway [67].

BAK effectiveness is highly dependent on concentration (dose relevance) and its ability to penetrate biological barriers (bioavailability) [67,71]. Research indicates that BAK concentrations between 0.5% and 2.0% are ideal for achieving visible anti-aging and anti-inflammatory benefits without the irritation typically associated with retinoids. At lower concentrations (e.g., 10 µg/mL), bakuchiol has been shown to reduce 5-α-reductase expression by approximately 40%, comparable to retinoic acid. Bakuchiol exhibits strong dose-dependent antibacterial activity. Its Minimum Inhibitory Concentration (MIC) is notably low for several pathogens: Staphylococcus aureus: 1 µg/mL, Staphylococcus epidermidis: 2 µg/mL, Candida albicans: 1.5 µg/mL. Bakuchiol has a high Log p value (6.1), making it practically insoluble in water. This necessitates advanced delivery systems for effective practical application. To overcome low bioavailability, researchers utilize various carrier systems such as nanomaterials.

BAK has demonstrated anti-breast cancer activity via S-phase arrest and apoptosis in experimental models. Although often promoted as a well-tolerated “natural retinol,” its estrogenic and anti-androgenic activities may produce off-target effects in hormone-sensitive patients, depending on concentration and tissue exposure. Additionally, its inhibition of multiple signaling kinases (including Hck, Blk, and p38 MAPK) suggests a broader biological activity beyond purely retinol-like effects, warranting cautious interpretation.

Particularly important are the breast cancer-specific data indicating that BAK interacts with estrogen receptors in a manner distinct from classical ERα agonists. Although it exhibits phytoestrogenic properties, experimental evidence in MCF-7 cells confirms it does not stimulate the proliferation of ERα-positive cells; instead, it shifts the signaling balance toward the antiproliferative activity of ERβ. This selective modulation resembles the action of SERMs with respect to its mechanism of action and may be associated with a distinct safety profile. Moreover, the ability of BAK to induce S-phase cell cycle arrest through activation of the ATM/ATR–Chk1/Chk2 axis—a mechanism demonstrated in breast cancer models —aligns with the effects of compounds that elicit replicative stress [42,43,44,45].

In vitro effects of bakuchiol often occur at concentrations between 1 µM and 200 µM. While these levels are high for systemic drug targets, they are highly relevant for topical skincare due to the high local concentrations achieved during application. Before considering information on bakuchiol, it is important to understand that the following information is for general knowledge and should not be taken as medical advice. Consult with a healthcare provider before making any decisions about your health or treatment. The relevance of micromolar in vitro data for bakuchiol is supported by the high concentrations used in clinical topical formulations. A standard bakuchiol cream at a clinically relevant concentration contains a high concentration of bakuchiol. Even if skin penetration is low, a fraction of a topical dose reaching the epidermis or dermis can still exceed the threshold required for biological effects such as collagen stimulation or antioxidant activity. Encapsulation technologies are sometimes used to help bakuchiol remain within the target skin layers rather than entering systemic circulation, which helps maintain high local concentrations. In vitro studies suggest that bakuchiol may offer various skin benefits and demonstrate a dose-dependent relationship for these effects. Significant protection against lipid peroxidation is observed at certain concentrations. Complete growth inhibition of certain oral bacteria has been observed at specific concentrations. Upregulation of collagen genes and inhibition of inflammatory markers typically occur in the micromolar range, which is consistent with the results seen with some topical applications. Cellular toxicity in some models has been observed to begin at certain concentrations. For topical use, this may be less of a concern as systemic absorption is generally minimal compared to the local application. Clinical trials often use specific concentrations of bakuchiol, which have shown potential to improve wrinkles, hyperpigmentation, and skin elasticity. These concentrations are specifically chosen to help bridge the gap between in vitro requirements and dermal delivery [67].

Critically, bakuchiol is a trendy, plant-derived retinol alternative praised for less irritation but suffers from limited, methodologically weak clinical studies, lacking robust evidence for its anti-aging claims, though it shows promise as an antioxidant and for sensitive skin; however, concerns remain about its low solubility, potential phototoxicity from natural sources (psoralens), and the need for better research to solidify its efficacy and safety, especially regarding potential phytoestrogen effects at high doses, as current praise outpaces scientific backing [5].

A key aspect of BAK’s activity is also its capacity to induce mitochondrial apoptosis, involving ROS accumulation, modulation of the Bax/Bcl-2 ratio, mitochondrial membrane depolarization and caspase activation. This mechanism is consistent with therapeutic strategies used in many classical anticancer drugs. Of particular note is the influence of BAK on BCSCs, which contribute to disease relapse, treatment resistance and enhanced metastatic potential. In these models, BAK limits its self-renewal capacity, reduces ALDH activity, and initiates BNIP3- and DAPK2-dependent apoptosis, while simultaneously inhibiting the TGF-β–Notch3–FASN axis, which is essential for EMT and the acquisition of a metastatic phenotype in breast cancer. Additionally, the development of BAK derivatives, such as BAK aminoguanidine or compound 19, confirms the high structural potential of this molecule for designing novel compounds with improved selectivity and potency. These derivatives intensify mitochondrial destabilization, amplify ROS-dependent apoptosis, and reduce invasion-related processes by inhibiting MMP-2 and MMP-9 and increasing E-cadherin expression, suggesting that BAK may serve as a valuable scaffold for further chemical modifications. Despite these promising results, it is important to emphasize the limitations of the current body of research. Most data come from in vitro studies, often conducted on a limited number of cell lines, which cannot fully replicate the complexity of the in vivo tumor microenvironment. Another challenge is the poor solubility and extensive first-pass metabolism of natural BAK, which may limit its bioavailability. There is also a lack of translational and clinical studies confirming its safety and efficacy in patients with breast cancer. These limitations clearly highlight potential directions for future research. Priority should be given to in vivo pharmacokinetic and pharmacodynamic studies to assess the bioavailability, metabolic stability and toxicity of BAK and its derivatives. Equally important will be the development of modern delivery systems such as nanoparticles, liposomal carriers or prodrugs aimed at improving bioavailability and enabling systemic administration. Further studies should also examine the effects of BAK on various breast cancer subtypes, including HER2-positive, luminal and particularly challenging TNBC, in order to precisely define its spectrum of activity. Another crucial area of investigation is the influence of BAK on the tumor microenvironment, especially interactions with immune cells, fibroblasts and the tumor stroma, as its anti-inflammatory and antioxidant properties may modulate key processes regulating tumor growth and metastasis. Given the pronounced effects of BAK on cancer stem cells, expanding research to models of minimal residual disease (MRD) and therapy-resistant cell populations appears especially promising. Furthermore, due to BAK’s influence on pathways associated with resistance and EMT, it is reasonable to explore its potential synergy with hormonal therapy, chemotherapy or inhibitors of PI3K/AKT, PARP and CDK4/6 [67].

The interplay between these systems operates through three primary stages: sensing and initiation (oxidative stress), pathway activation and crosstalk (MAPK and NF-κB), and execution (mitochondrial apoptosis).

Consolidating oxidative stress, mitochondrial apoptosis, and NF-κB/MAPK signaling into a unified framework reveals an interconnected network of stress sensing, inflammatory feedback, and cell fate determination. In this integrated model, reactive oxygen species (ROS) act as both the initial trigger and the central signal amplifier across these pathways [72].

## 5. Conclusions

Available evidence indicates that bakuchiol is a biologically versatile compound with significant potential in the context of breast cancer therapy. Its ability to modulate oxidative stress, mitochondrial function, apoptosis, estrogen receptor signaling, and pathways associated with proliferation, metastasis, and treatment resistance positions it as a promising candidate for further oncological investigation. Importantly, BAK demonstrates activity against both differentiated tumor cells and breast cancer stem cells, which are critical drivers of recurrence and therapeutic failure. Despite these encouraging findings, current knowledge remains a synthesis of general pharmacological data and initial breast cancer-specific studies, predominantly conducted in vitro. The distinction between universal mechanisms (e.g., DNA polymerase inhibition) and those specifically validated in breast models (e.g., ERβ-mediated antiproliferation) is critical for future translational success. The pharmacokinetic limitations of natural bakuchiol further underscore the need for optimized delivery strategies and the development of derivatives with improved stability and bioavailability. Future research should focus on comprehensive preclinical evaluation, assessment across diverse breast cancer subtypes, and investigation of potential synergistic effects with established therapies. Overall, bakuchiol represents a promising natural molecule with meaningful mechanistic foundations for anticancer activity, yet its translation into clinical practice will require rigorous, multidimensional research to fully validate these extrapolated mechanisms in human breast cancer patients.

## Figures and Tables

**Table 1:** Overview of Plants Serving as Sources of Bakuchiol.

Plant Name	References
*Prosopis glandulosa*	[7]
*Otholobium pubescens*	[8]
*Ulmus davidiana*	[9]
*Pimelea drupacea*	[10]
*Piper longum*	[11]
*Fructus psoraleae*	[12]
*Aerva sanguinolenta*	[13]
*Nepeta angustifolia*	[14]
*Spiraea formosana*	[15]
*Psoralidium tenuiflorum*	[16]
*Bridelia retusa*	[17]
*Elaeagnus bockii*	[18]

**Table 2:** Compilation of the most significant achievements in the natural extraction of BAK.

**Plant Species**	**Brief Description**	**Reference**
*Psoralidium tenuiflorum*	The whole plants were extracted with ethyl acetate, and the resulting extract was evaporated. The extract was then partitioned between hexane and aqueous methanol. The methanolic aqueous phase was diluted with water and extracted with dichloromethane, yielding bioactive dichloromethane and methanolic fractions, as well as an inactive hexane fraction. The bioactive fractions were combined and chromatographically separated using gradient elution with methanol in water, followed by ethyl acetate in hexane. The most active ethyl acetate fraction was finally purified by reversed-phase liquid chromatography using aqueous methanol.	[16]
*P. corylifolia*	The fruits were extracted with 95% ethanol under reflux, and the resulting extract was evaporated to dryness. The ethanol extract was then successively partitioned into n-hexane, dichloromethane, ethyl acetate, and n-butanol. The hexane fraction was subjected to column chromatography, initially eluted with a hexane–ethyl acetate mixture (9:1), followed by methanol and water (7:3).	[30]
*P. corylifolia*	Fruit samples were subjected to ultrasonic extraction with methanol acidified with concentrated hydrochloric acid (5:1). Extraction was carried out at 20 °C for 45 min, after which the mixture was left at room temperature for 30 min. The sample was then centrifuged at 3000× *g* for 20 min, and the collected supernatant was diluted with the extraction solution and stored at 4 °C.	[31]
*P. glandulosa*	The aerial parts of the plants were briefly extracted with dichloromethane by immersing the sample in the solvent for 30 s at room temperature. The resulting solution was filtered and concentrated under reduced pressure. The resinous extract was purified chromatographically using an ethyl acetate–hexane mixture for elution.	[32]
*P. corylifolia*	The fruits were extracted sequentially with n-hexane, ethyl acetate, and methanol. The ethyl acetate fraction was then subjected to chromatographic separation, and the obtained components were purified using HPLC and TLC methods.	[33]
*P. corylifolia* L.	The fruits were extracted with methanol for 3 days, and the resulting extract was concentrated under reduced pressure at 35 °C. The residue was partitioned between ethyl acetate and water (1:1), after which the ethyl acetate–soluble fraction was subjected to repeated chromatography using an ethyl acetate–hexane mixture for elution. Final purification was performed by preparative TLC.	[34]
*P. corylifolia*	The fruits were subjected to ultrasonic extraction with ethanol for 30 min, after which the solution was filtered and cooled to room temperature. The resulting extract was dried, concentrated under reduced pressure at 50 °C, and dissolved in methanol. The extract was then separated using HSCCC with a biphasic n-hexane–ethyl acetate–methanol–water system (5:5.5:6.5:5, *v*/*v*/*v*/*v*).	[35]
*P. corylifolia*	The seeds were extracted using supercritical fluid extraction with pure CO_2_ at 280 bar and 40 °C, with a flow rate of 4 L NPT/min (3.6 g/min). Extraction was carried out in static–dynamic cycles (10 min static, 20 min dynamic) for a total duration of 330 min. The resulting extract was stored in the dark at −20 °C	[36]

**Table 3:** Information on inflammation, angiogenesis, cell cycle, apoptosis and autophagy in BAK in breast cancer treatment.

Signaling Pathway	Main Molecular Targets/Pathways	Mechanism of Action of Bakuchiol	Relevance to Breast Cancer
Inflammation [65]	p38 MAPK, ERK, TLR4/NF-κB, IκBα, p65	Inhibition of NF-κB activation through suppression of IκBα and p65 phosphorylation; downregulation of p38 MAPK/ERK and TLR4/NF-κB signaling pathways	Reduced production of pro-inflammatory cytokines (TNF-α, IL-6), decreased expression of iNOS and COX-2; attenuation of the pro-inflammatory tumor microenvironment that promotes breast cancer progression
Angiogenesis [66]	(No clearly defined direct targets)	Direct effects on angiogenic signaling pathways have not been clearly defined; potential indirect modulation via anti-inflammatory and antiproliferative activities	Possible indirect inhibition of tumor neovascularization through reduction of inflammatory and proliferative signals supporting angiogenesis
Cell cycle [67]	p53, p21, p27, CDK2, CDK4	Induction of cell cycle arrest via upregulation of cyclin-dependent kinase inhibitors (p53, p21, p27) and downregulation of CDK2 and CDK4	Inhibition of G1 to S phase transition, leading to suppressed proliferation of breast cancer cells
Apoptosis [68]	Caspase-3, Bax/Bcl-2, tBid/Bid, JNK	Activation of caspase-3-dependent apoptosis; increased Bax/Bcl-2 and tBid/Bid ratios; activation of the JNK pathway and mitochondrial translocation of Bax	Induction of mitochondrial apoptosis in cancer cells, contributing to the elimination of breast cancer cells
Autophagy [66,67]	AMPK, Akt/mTOR	Activation of AMPK accompanied by inhibition of the Akt/mTOR pathway, a negative regulator of autophagy	Induction of autophagy, which may suppress breast cancer cell growth and survival and enhance anticancer effects

**Table 4:** Comparative summarized information on breast cancer cell lines used, ER/HER2 status, bakuchiol concentrations and primary outcomes [67].

Cell Line	ER Status	HER2 Status	Bakuchiol Concentration	Primary Outcomes
MCF-7	Positive (ERα+)	Negative	Low dose (<2 μg/mL): Stimulates proliferation. High dose (>2 μg/mL): Inhibits growth.	Biphasic effect: Pro-proliferative at low doses; antiproliferative and pro-apoptotic at high doses via mitochondrial pathway.
MDA-MB-231	Negative (Triple-negative)	Negative	0–10 μg/mL (IC50: 8.9–13.1 μg/mL)	S-phase arrest: Dose-dependent growth inhibition and cell cycle arrest.
BCSCs (from MCF-7)	Positive	Negative	Not explicitly specified in snippet (often similar to high-dose MCF-7 studies)	Inhibition of stemness: Suppressed mammosphere formation, induced apoptosis, and inhibited in vivo metastasis in zebrafish.
A549/HT29/MCF7	Mixed	Mixed	Not specified in snippet	Anti-metastatic: Inhibited epithelial–mesenchymal transition (EMT) and lung metastasis in animal models.

## Data Availability

Not applicable.
